# Apolipoprotein A-IV concentrations and cancer in a large cohort of chronic kidney disease patients: results from the GCKD study

**DOI:** 10.1186/s12885-024-12053-8

**Published:** 2024-03-07

**Authors:** Barbara Kollerits, Simon Gruber, Inga Steinbrenner, Johannes P. Schwaiger, Hansi Weissensteiner, Sebastian Schönherr, Lukas Forer, Fruzsina Kotsis, Ulla T. Schultheiss, Heike Meiselbach, Christoph Wanner, Kai-Uwe Eckardt, Florian Kronenberg, for the GCKD Investigators, Markus P. Schneider, Mario Schiffer, Hans-Ulrich Prokosch, Barbara Bärthlein, Andreas Beck, André Reis, Arif B. Ekici, Susanne Becker, Ulrike Alberth-Schmidt, Anke Weigel, Sabine Marschall, Eugenia Schefler, Gerd Walz, Anna Köttgen, Ulla T. Schultheiß, Simone Meder, Erna Mitsch, Ursula Reinhard, Jürgen Floege, Turgay Saritas, Alice Gross, Elke Schaeffner, Seema Baid-Agrawal, Kerstin Theisen, Hermann Haller, Martin Zeier, Claudia Sommerer, Mehtap Aykac, Gunter Wolf, Martin Busch, Andy Steiner, Thomas Sitter, Vera Krane, Antje Börner-Klein, Britta Bauer, Peter Oefner, Wolfram Gronwald, Matthias Schmid, Jennifer Nadal

**Affiliations:** 1 Institute of Genetic Epidemiology, Medical University of Innsbruck, Schöpfstraße 41, Innsbruck, 6020 Austria; 2 Institute of Genetic Epidemiology, Faculty of Medicine and Medical Center - University of Freiburg, Freiburg, Germany; 3 Department of Nephrology and Hypertension, University Hospital Erlangen, Friedrich-Alexander-Universität Erlangen-Nürnberg, Erlangen, Germany; 4 German Chronic Kidney Disease Study, Erlangen, Germany; 5 Department of Medicine IV – Nephrology and Primary Care, Faculty of Medicine and Medical Center - University of Freiburg, Freiburg, Germany; 6 Division of Nephrology, Department of Internal Medicine I, University Hospital Würzburg, Würzburg, Germany; 7 Department of Nephrology and Medical Intensive Care, Charité – Universitätsmedizin Berlin, Berlin, Germany

**Keywords:** Apolipoprotein A-IV, Kidney function, Cancer, Inflammation, Prospective study

## Abstract

**Background:**

Chronic kidney disease (CKD) is highly connected to inflammation and oxidative stress. Both favour the development of cancer in CKD patients. Serum apolipoprotein A-IV (apoA-IV) concentrations are influenced by kidney function and are an early marker of kidney impairment. Besides others, it has antioxidant and anti-inflammatory properties. Proteomic studies and small case–control studies identified low apoA-IV as a biomarker for various forms of cancer; however, prospective studies are lacking. We therefore investigated whether serum apoA-IV is associated with cancer in the German Chronic Kidney Disease (GCKD) study.

**Methods:**

These analyses include 5039 Caucasian patients from the prospective GCKD cohort study followed for 6.5 years. Main inclusion criteria were an eGFR of 30–60 mL/min/1.73m^2^ or an eGFR > 60 mL/min/1.73m^2^ in the presence of overt proteinuria.

**Results:**

Mean apoA-IV concentrations of the entire cohort were 28.9 ± 9.8 mg/dL (median 27.6 mg/dL). 615 patients had a history of cancer before the enrolment into the study. ApoA-IV concentrations above the median were associated with a lower odds for a history of cancer (OR = 0.79, *p* = 0.02 when adjusted age, sex, smoking, diabetes, BMI, albuminuria, statin intake, and eGFR_creatinine_). During follow-up 368 patients developed an incident cancer event and those with apoA-IV above the median had a lower risk (HR = 0.72, 95%CI 0.57–0.90, *P* = 0.004). Finally, 62 patients died from such an incident cancer event and each 10 mg/dL higher apoA-IV concentrations were associated with a lower risk for fatal cancer (HR = 0.62, 95%CI 0.44–0.88, *P* = 0.007).

**Conclusions:**

Our data indicate an association of high apoA-IV concentrations with reduced frequencies of a history of cancer as well as incident fatal and non-fatal cancer events in a large cohort of patients with CKD.

**Graphical Abstract:**

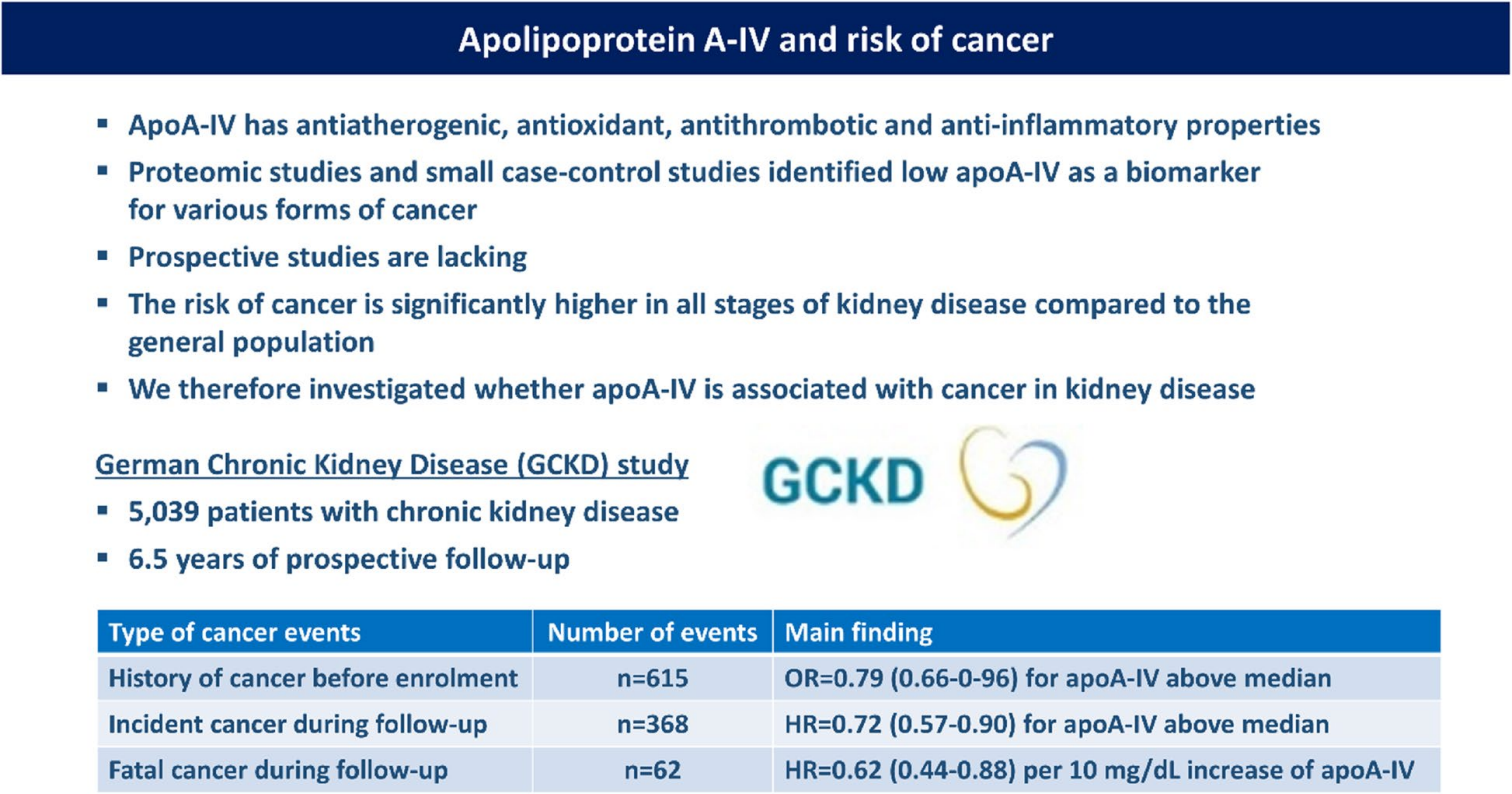

**Supplementary Information:**

The online version contains supplementary material available at 10.1186/s12885-024-12053-8.

## Introduction

Chronic kidney disease (CKD) is a common disease, resulting in about 1–2 million deaths per year worldwide and is expected to be the fifth leading cause of death by 2040. Apart from the risk of kidney failure, CKD is associated with an increased cardiovascular morbidity and mortality [1].

The second leading cause of death worldwide is cancer [2]. The risk of cancer is significantly higher in all stages of kidney disease compared to the general population [3, 4]: there are multifactorial causes for this increased risk depending on cancer site, cause of CKD or immune dysregulation caused by chronic uraemia. Some cancer treatments also have a negative influence on kidney function [2, 3]. Systemic inflammation and oxidative stress are both conditions highly connected to kidney dysfunction [5] and can promote the development of cancer [6]. Furthermore, an insufficient symptom recognition in CKD patients results in a delayed cancer diagnosis at more advanced stages with an increased cancer mortality. Therefore early detection is essential to improve cancer outcomes in these patients [4, 7].

Apolipoprotein A-IV (apoA-IV) is a 46 kDa glycoprotein mainly produced in enterocytes of the small intestine [8]. It serves as one of the structural proteins of chylomicrons, VLDL and HDL or is found to be unbound to lipoproteins [9–11]. ApoA-IV binds to cells, promotes cholesterol efflux and is involved in reverse cholesterol transport [12]. It has antiatherogenic, antioxidant as well as antithrombotic and anti-inflammatory properties [13–16]. For example, experimental mouse models demonstrated that apoA-IV significantly modified and attenuated inflammation [14, 15].

The concentrations of apoA-IV are markedly increased in CKD, particularly in patients with kidney failure on dialysis [13]. ApoA-IV is filtered by the glomerulus and reabsorbed by proximal tubular cells [17]. It was found to be an early marker of kidney impairment and to predict the progression of CKD [18, 19]. Moreover, low apoA-IV was found to be associated with cardiovascular disease in the non-CKD population [20, 21] as well as with increased mortality and cardiac outcomes in CKD patients [22, 23]. The increase of apoA-IV in CKD patients who have overall a high cardiovascular disease rate is at the first glance counterintuitive. However, the increase is explained by an impaired filtration capacity and it might counteract the high oxidative stress and inflammatory burden of these patients.

Several proteomics studies identified low apoA-IV to be associated with various forms of cancer (Supplementary Table 1). Only few of them validated these findings by quantitative methods such as ELISA and if so, it has been done in small case–control studies [24–31] but no prospective studies. We therefore analysed in the prospective German Chronic Kidney Disease (GCKD) study whether serum apoA-IV concentrations are associated with a history of cancer as well as incident cancer events in this cohort of CKD patients at high risk for cancer.

## Materials and methods

### German Chronic Kidney Disease (GCKD) study

The GCKD study includes 5217 carefully phenotyped Caucasian CKD patients recruited between 2010 and 2012 who were under regular care by nephrologists. It is an ongoing multicentre prospective cohort study. The study design and detailed characteristics have been published [23, 32, 33]. The study aimed to enrol patients with mild to severe CKD mostly in stage G3: that means an estimated glomerular filtration rate (eGFR) of 30–60 mL/min/1.73m^2^ (KDIGO stage G3, A1-3) or an eGFR of > 60 mL/min/1.73m^2^ in the presence of overt albuminuria defined by a urine albumin creatinine ratio (UACR) of > 300 mg/g or equal amounts of proteinuria (KDIGO stage G1-2, A3). Non-Caucasian ethnicity, solid organ or bone marrow transplantation, active malignancy within 24 months before screening, New York Heart Association stage IV heart failure, legal attendance, or inability to provide consent were exclusion criteria. At baseline, blood and urine samples were collected by trained personnel, processed, and sent on dry ice to a central biobank. Routine laboratory parameters were measured centrally [33]. eGFR was estimated using the CKD-EPI formula [34].

Each participant provided written informed consent, and all methods were performed in accordance with approved guidelines and the Declaration of Helsinki. The study was approved by the ethics committees of each study center and registered in the national registry for clinical studies (DRKS 00003971). Data are collected and managed using Askimed (https://www.askimed.com) as a cloud-based web platform.

Patients are followed on a yearly basis by trained personnel who collect data on hospitalisations, outcome events and recent medical history using a structured interview. Any hospital discharge reports are collected from the treating physicians and/or hospitals. Endpoints are continually extracted from these reports by an endpoint committee.

### Definition of outcomes

Incident cancer events included non-fatal and fatal cancer events. They were categorised in the groups renal tract cancers (kidney, bladder, urothelial cancer), male cancers (prostate cancer, others), female cancers (e.g. breast, cervix, uterus, ovary), cancer of digestive system (oesophagus, stomach, small intestine, colon, rectum), skin cancer (melanoma, squamous cell carcinoma but no basal cell carcinoma), lung cancer, haematological cancers (lymphoma, leukaemia, multiple myeloma, and other malignant haematological conditions), cancers of the abdominal solid organs (liver, gallbladder, biliary tract, pancreas, other digestive organs), other cancers and cancers of unknown origin. Incident cancer events were events newly occurring during follow-up excluding those with a history of cancer (i.e. type of cancer that occurred the first time more than 24 months before the screening for the study). For the present analyses, prospectively collected endpoints are taken from the 6.5-year follow-up with data export from October 8, 2020. Details and frequencies of these main cancer groups are given in Supplementary Table 2. Fatal cancer events were investigated as an additional endpoint.

History of cancer more than two years before the enrolment into the study was collected by interview and patient discharge information and the retrospective collection of this information had not the level of standardization as the collection of the incident events.

### Measurement of apoA-IV serum concentrations

Serum apoA-IV concentrations were measured at the Medical University of Innsbruck with a double-antibody ELISA using an affinity-purified polyclonal rabbit anti-human apoA-IV antibody for coating. This antibody coupled to horseradish peroxidase was used for detection. Serum with a known content of apoA-IV served as the calibration standard. Patient samples were diluted 1:12500. In case of the rare situation of very low or very high concentrations outside of the linear range, measurements were repeated using a lower or higher dilution, respectively. The intra- and inter-assay coefficients of variation were 2.7% and 6%, respectively [19, 22, 35, 36].

### Statistical analysis

Baseline characteristics of GCKD study participants are provided for the patient strata non-cancer, prevalent cancer (those with a history of cancer before enrolment), and incident cancer and by quartiles of apoA-IV concentrations for the total group. Jonckheere-Terpstra tests for trend were performed for comparison of continuous variables between quartile groups of apoA-IV. Linear by linear χ^2^-square tests were applied for comparison of categorical variables. The association of apoA-IV concentrations with history of cancer was examined by logistic regression analyses. Cox proportional hazard regression models were used to calculate cause-specific hazard ratios (HR) with corresponding 95% confidence intervals for incident and fatal cancer endpoints. All deaths other than cancer were treated as censored observations. To prove the proportional hazards assumption a χ^2^-test based on Schoenfeld residuals was performed. Subdistribution hazard ratios (SHR) based on competing risks survival regression were calculated additionally treating all other causes of death as competing events. For both baseline and follow-up analyses, model 1 was adjusted for age, sex, eGFR and UACR. Model 2 additionally included statin use, smoking, BMI, diabetes (patients with a history of cancer were excluded in the Cox regression analyses). This selection was based on clinical reasons and by taking into account differences of variables between quartiles of apoA-IV as shown in Supplementary Table 3. In early CKD, increased risk of cancer incidence and death was reported to be better predicted by eGFR based on cystatin C (eGFRcys) than eGFR based on creatinine (eGFRcrea) or a combination of creatinine and cystatin C [4]. Thus, eGFRcys was used in all models of follow-up analyses. As this was not shown for history of cancer, we additionally provide adjustment for eGFRcrea for baseline analyses. UACR and high sensitivity C reactive protein (hs-CRP) were log-transformed based on the natural logarithm (ln) due to their skewed distribution. As CRP is not deemed to have a causal role in cancer [37] we additionally adjusted for hs-CRP only in sensitivity analyses.

Analyses were reported for quartile groups of apoA-IV, where quartile 1 was the reference, as well as for an increase of 10 mg/dL in apoA-IV concentration. The increment of 10 mg/dL is almost identical to the standard deviation of 9.8 mg/dL of the entire cohort. Based on visual inspection of quartile groups of apoA-IV for baseline and follow-up, further analyses were performed: where appropriate, patient values were compared above and below the median of apoA-IV concentrations.

Statistical analysis was performed using SPSS for Windows, version 29 (IBM Corp., Armonk, New York, NY, USA) and R for Windows, version 4.3.1 (Vienna, Austria) (https://www.r-project.org). For all analyses performed, a two-sided test *P*-value < 0.05 was considered statistically significant.

## Results

### Baseline characteristics

Five thousand thirty-nine of five thousand two hundred seventeen patients included in the GCKD study had complete baseline data on age, sex, eGFRcys, UACR, apoA-IV concentrations, cancer information and follow-up data available. The mean serum concentration of apoA-IV was 28.9 ± 9.8 mg/dL (median 27.6 mg/dL). Table 1 shows clinical characteristics at baseline (at the time of enrolment) for non-cancer patients (did not have cancer before enrolment and also not during follow-up), prevalent cancer patients (those with a history of cancer before enrolment), and incident cancer patients (those who developed a new cancer event during follow-up which has not been experienced before enrolment). The table provides data on apoA-IV concentrations, age, sex, kidney function parameters, body mass index, smoking status, diabetes mellitus, lipids, hs-CRP and statin intake. Supplementary Table 3 shows further baseline characteristics by quartiles of apoA-IV in the total cohort.

**Table 1:** Baseline characteristics of German Chronic Kidney Disease (GCKD) study patients stratified by cancer status^a^

	**Non-cancer (*n* = 4056)**^**b**^	**Prevalent cancer (*n* = 615)**^**b**^	**Incident cancer (*n* = 368)**^**b**^
ApoA-IV (mg/dL)	29.0 ± 9.9 [22.0; 27.9; 34.4]	28.1 ± 9.1 [21.6; 26.4; 33.0]	28.7 ± 10.1 [22.0; 27.1; 33.9]
Age (years)	59 ± 12 [51; 62; 69]	65 ± 9 [60; 68; 71]	65 ± 9 [62; 68; 71]
Female sex, n (%)	1653 (40.8%)	246 (40.0%)	106 (28.8%)
eGFR (mL/min/1.73m^2^)^c^	51 ± 20 [36; 47; 61]	48 ± 18 [35; 46; 59]	45 ± 17 [33; 42; 54]
UACR (mg/g)^d^	448 ± 962 [10; 55; 422]	365 ± 1035 [7; 30; 210]	343 ± 782 [10; 49; 286]
Body mass index, (kg/m^2^)	29.7 ± 6.0 [25.6;28.8;33.1]	29.9 ± 5.7 [25.8;29.1;33.2]	30.5 ± 5.5 [27.2;29.6;33.8]
Hs-CRP, (mg/L)	4.6 ± 8.0 [1.0; 2.2; 4.9]	5.3 ± 8.8 [1.2; 2.5; 5.7]	5.4 ± 10.1 [1.4; 2.7; 5.7]
LDL cholesterol, (mg/dL)	119 ± 44 [90; 115; 143]	116 ± 43 [86; 112; 139]	113 ± 41 [84; 109; 142]
HDL cholesterol, (mg/dL)	52 ± 18 [40; 49; 62]	51 ± 19 [38; 47; 61]	50 ± 18 [38; 45; 59]
Triglycerides, (mg/dL)	199 ± 129 [118; 168; 239]	202 ± 120 [116; 169; 245]	202 ± 133 [120; 170; 246]
Smoker and ex-smoker, n (%)	2354 (58.0%)	373 (60.7%)	239 (65.0%)
Diabetes, n (%)	1383 (34.1%)	241 (39.2%)	162 (44.0%)
Statin use, n (%)	1918 (47.3%)	273 (44.4%)	204 (55.4%)

Data are provided as mean ± SD [25th, 50^th^ (median) and 75th percentile or number (%), (percentage considering missing values). Hs-CRP and urine-albumin values that were below the lower detection limit (LOD) were replaced by LOD/√2. BMI was corrected for amputation

^a^Study participants are stratified by non-cancer patients (did not have cancer before enrolment and also not during follow-up), prevalent cancer patients (those with a history of cancer before enrolment), and incident cancer patients (those who developed a new cancer event during follow-up which has not been experienced before enrolment)

^b^Number of patients based on those with available data on age, sex, eGFRcys, urine albumin-creatinine ratio (UACR), apolipoprotein A-IV measurements, cancer information and censoring date

^c^eGFR denotes glomerular filtration rate calculated according to the CKD-EPI equation based on cystatin C [38]

^d^UACR was calculated according to the following equation: albumin in urine (mg/l) × 100 / creatinine in urine (mg/dl) and is given in mg/g

### History of cancer

At baseline, 615 patients with available data on apoA-IV, age, sex, and kidney function, had already experienced a cancer event more than two years before the enrolment (active malignancy within 24 months before screening was an exclusion criterion). First, an analysis based on quartiles of apoA-IV concentrations was done with quartile 1 as reference group. Results of both model 1 and model 2 showed in quartiles 3 and 4 a lower odds for history of cancer when compared to patients in quartile 1 and 2. When we compared patients above and below the median of apoA-IV concentrations we barely missed significance in terms of a lower odds for a history of cancer in patients above the median of apoA-IV with an OR = 0.84 (95% CI 0.70–1.02, *p* = 0.07) (Table 2). When adjusting for eGFRcrea, estimates reached significance (OR = 0.79 (95% CI 0.66–0.96, *p* = 0.02) (Table 2).

**Table 2:** Association of apolipoprotein A-IV with history of cancer (615 and 611 out of 5039 patients). Results are given for both eGFR adjustments (once calculated with cystatin C and once with creatinine)

	**eGFR**_**cystatin-C**_** adjusted**			**eGFR**_**creatinine**_** adjusted**		
	**OR**	**95% CI**	***p*-value**	**OR**	**95% CI**	***p*-value**
**Calculations for median of apoA-IV concentrations**^a^						
Model 1	0.85	0.71–1.03	0.09	0.81	0.67–0.97	0.02
Model 2	0.84	0.70–1.02	0.07	0.79	0.66–0.96	0.02
**Calculations per quartile of ApoA-IV concentrations**						
**Model 1**						
Quartile 1: 163 cases^b^	1.00			1.00		
Quartile 2: 174 cases^b^	1.07	0.85–1.35	0.58	1.03	0.82–1.31	0.79
Quartile 3: 147 cases^b^	0.89	0.69–1.14	0.36	0.84	0.65–1.07	0.16
Quartile 4: 131 cases^b^	0.88	0.67–1.15	0.35	0.80	0.61–1.06	0.12
**Model 2**						
Quartile 1: 163 cases^b^	1.00			1.00		
Quartile 2: 172 cases^b^	1.05	0.83–1.33	0.69	1.01	0.80–1.28	0.92
Quartile 3: 144 cases^b^	0.87	0.68–1.12	0.27	0.81	0.63–1.05	0.11
Quartile 4: 128 cases^b^	0.86	0.65–1.14	0.29	0.78	0.59–1.04	0.09

Model 1: adjusted for age, sex, ln-urine albumin-creatinine ratio, eGFR_cystatin C_ or eGFR_creatinine_

Model 2: as model 1 plus statin use, smoking, BMI, diabetes

*Abbreviations*: *OR* odds ratio, *CI* confidence interval

^a^Reference category includes apoA-IV values below median. The median apoA-IV concentration is 27.6 mg/dL (referring to the total group of 5039 patients). There are 278 cases above, and 337 cases below the median (model 1), and 272 cases above, and 335 cases below the median (model 2). Small differences in the number of cases between eGFR_cystatin C_ (*n* = 615) and eGFR_creatinine_ (*n* = 611) adjustment are caused due to few more missing variables for eGFR_creatinine_

^b^“Cases” refers to the number of patients with history of cancer. Differences in number of cases between model 1 and 2 are explained by patients with few missing covariates for model 2

### Incident cancer

During a median follow-up of 6.5 years, 368 patients experienced a new incident cancer event which has not been experienced before enrolment (a relapse of a given cancer did not count for this analysis). There was no violation of the proportional hazards assumption. An analysis based on quartiles of apoA-IV concentrations was performed with quartile 1 as reference group. Results of model 1 and the fully adjusted model 2 indicated that especially patients in quartiles 3 and 4, although missing significance in quartile 4 (*p* = 0.07), had a lower risk for incident cancer (Table 3). Based on this observation, risk estimates above and below the median of apoA-IV concentrations were compared. This revealed a significantly lower risk for patients with apoA-IV concentrations above the median in the fully adjusted model 2 (HR = 0.72, 95%CI 0.57–0.90, *p* = 0.004).

**Table 3:** Association of apolipoprotein A-IV with incident cancer during the prospective follow-up in patients without a history of cancer at the baseline investigation (4424 out of 5039 patients) ^a^

		**HR**	**95% CI**	***p*-value**
**Calculations for median of apoA-IV concentrations**^b^				
Model 1: 368 cases (174 above and 194 below median)^c^		0.73	0.58–0.91	0.006
Model 2: 362 cases (171 above and 191 below median)^c^		0.72	0.57–0.90	0.004
**Calculations per quartile of ApoA-IV concentrations**				
Model 1	Quartile 1 (90 cases)^c^	1.00		
	Quartile 2 (104 cases)^c^	1.00	0.75–1.33	0.98
	Quartile 3 (85 cases)^c^	0.73	0.54–0.99	0.04
	Quartile 4 (89 cases)^c^	0.74	0.53–1.02	0.07
Model 2	Quartile 1 (88 cases)^c^	1.00		
	Quartile 2 (103 cases)^c^	1.02	0.76–1.36	0.91
	Quartile 3 (83 cases)^c^	0.72	0.53–0.98	0.04
	Quartile 4 (88 cases)^c^	0.73	0.53–1.02	0.07

Model 1: adjusted for age, sex, ln-urine albumin-creatinine ratio and eGFR_cystatin-C_

Model 2: as model 1 plus statin use, smoking, BMI, diabetes

*Abbreviations*: *HR* hazard ratio, *CI* confidence interval

^a^Patients with a history of cancer at the time of enrollment were not considered in this analysis

^b^Reference category includes apoA-IV values below median. The median apoA-IV concentration is 27.6 mg/dL (referring to the total group of 5039 patients)

^c^“Cases” refers to the number of patients with incident cancer events. Differences in number of cases between model 1 and 2 are explained by patients with few missing covariates for model 2

### Fatal cancer

Of the 4424 patients with no history of cancer before enrolment into the study, 62 patients died from cancer during follow-up. The analysis based on quartiles of apoA-IV revealed lower risks for quartiles 2, 3 and 4, with statistical significance in quartile 2 and 4 and missing significance in quartile 3 (Table 4). Furthermore, each increase in apoA-IV concentrations of 10 mg/dL, was associated with a 40% lower risk to die when using the age-, sex- and kidney function-adjusted model 1 (HR = 0.60, 95%CI 0.43–0.85, *p* = 0.003). Model 2 additionally adjusting for statin use, diabetes, BMI, and smoking, revealed very similar results (HR = 0.62, 95%CI 0.44–0.88, *p* = 0.007).

**Table 4:** Association of apolipoprotein A-IV with fatal cancer during the prospective follow-up and without a history of cancer at the baseline investigation (4424 out of 5039 patients)^a^

		**HR**	**95% CI**	***p*-value**
**Calculations per 10 mg/dL increment of apoA-IV concentrations**				
Model 1: 62 cases^b^		0.60	0.43–0.85	0.003
Model 2: 60 cases^b^		0.62	0.44–0.88	0.007
**Calculations per quartile of ApoA-IV concentrations**				
Model 1	Quartile 1 (21 cases)^b^	1.00		
	Quartile 2 (11 cases)^b^	0.42	0.20–0.87	0.02
	Quartile 3 (19 cases)^b^	0.60	0.32–1.14	0.12
	Quartile 4 (11 cases)^b^	0.32	0.14–0.71	0.005
Model 2	Quartile 1 (19 cases)^b^	1.00		
	Quartile 2 (11 cases)^b^	0.46	0.22–0.98	0.04
	Quartile 3 (19 cases)^b^	0.65	0.34–1.26	0.21
	Quartile 4 (11 cases)^b^	0.35	0.15–0.79	0.01

Model 1: adjusted for age, sex, eGFR_cystatin-C_, ln-urine albumin-creatinine ratio

Model 2: as model 1 plus statin use, smoking, BMI, diabetes

*Abbreviations*: *HR* hazard ratio, *CI* confidence interval

^a^Patients with a history of cancer at the time of enrollment were not considered in this analysis

^b^“Cases” refers to the number of patients with fatal cancer events. Differences in number of cases between model 1 and 2 are explained by few patients with some missing covariates for model 2

### Additional analyses

We additionally adjusted the main models for HDL-cholesterol, LDL-cholesterol and triglyceride concentrations. These additional analyses did not change the association of apoA-IV with prevalent and incident cancer events which might be explained by the rather weak association of apoA-IV concentration with lipids (Supplementary Tables 4, 5 and 6).

For incident and fatal cancer, we analysed whether the association of apoA-IV with these outcomes changed when we adjusted for a history of cancer instead of excluding those cases. The hazard ratios were only minimally influenced by this approach (Supplementary Tables 7 and 8).

In a further sensitivity analysis we adjusted the baseline and all follow-up analyses additionally for hs-CRP, which only marginally changed the association between apoA-IV and cancer outcomes (Supplementary Tables 9, 10 and 11). However, adjustment for hs-CRP has to be considered with caution because the classical requirements for a confounder are not entirely fulfilled, since it has been shown by genetic studies that genetically explained hs-CRP concentrations are not significantly associated with cancer [37]. Furthermore, we did not adjust for socioeconomic status (education and income) since this status was not associated with apoA-IV concentrations.

The subdistribution hazard ratios for incident and fatal cancer outcomes were only slightly attenuated as compared to the cause-specific hazard ratios (Supplementary Tables 12 and 13).

## Discussion

We observed in this large cohort study of patients with mild to severe CKD i) that higher apoA-IV concentrations at baseline were associated with a lower odds for a history of cancer and ii) that during 6.5 years of follow-up higher apoA-IV concentrations were associated with a lower risk for incident cancer, particularly fatal cancer outcomes. All these associations were independent of kidney function. To our knowledge this is the largest and the only prospective study up to now which has investigated the association of apoA-IV with cancer, independent whether the literature in CKD or non-CKD patients is considered (Graphical Abstract).

Several proteomic studies have suggested apoA-IV as a biomarker and a tool for detection of various forms of cancer (Supplementary Table 1). Most of these studies investigated only a small number of serum or plasma samples and only few validated the results by an independent and quantitative method such as an ELISA [24–31]. Most data are available for ovarian cancer with quantitatively validated lower apoA-IV concentrations observed in three studies [24–26]. Further data were reported for pancreatic, oral, hepatocellular, and thyroid cancer (Supplementary Table 1). The distribution of cancer types in our GCKD study is comparable to what is reported for patients with moderate to severe CKD. Cancers of the urinary tract (kidney, bladder, urothelial cancer), prostate cancer, cancers of the digestive organs, skin cancers, cancer of the breast and female reproductive organs and lung cancers were among the most frequent cancers among these patients [4, 39, 40]. Whether apoA-IV is a biomarker of selected cancers or causally associated with cancer is currently not known.

### Possible explanations for the inverse association of apoA-IV with cancer

A number of biological entities constitute the so-called hallmarks of cancer, with inflammation being one of them [41] in turn increasing the probability for metastasis [42]. The known anti-inflammatory properties of apo-AIV [14, 15] and the link to endothelial function [43], a further component of the tumour microenvironment, could lead to the assumption, that ApoA-IV might be an important marker connected to several pathways in the development of cancer.

In patients with early stages of CKD the risk for cancer is already increased, in particular in males, and the risk increases gradually with more advanced stages of CKD [3]. Two cohort studies reported a stepwise association between severity of CKD and cancer mortality [3]. In turn, CKD is prevalent in approximately 12–25% of cancer patients [39]. Systemic inflammation and oxidative stress are common in patients with advanced CKD and increase with disease progression [44]. About 20–25% of all cancers are discussed to be triggered by chronic infection or inflammation. Several pathways, intracellular signaling cascades and related mechanisms were described that support this hypothesis such as DNA damage, cell damage, proliferation and transformation, immunosuppression, oxidative stress, angiogenesis or abnormal epithelial cell growth [45–47]. Thus, such inflammatory processes might be one of the main drivers for the higher risk for cancer in CKD, comparable to that for atherosclerosis [48].

To damage pathogens and protect the organism from harmful antigens, the body releases reactive oxygen species (ROS) in inflamed tissue [46]. Antioxidant processes prevent the accumulation of ROS and reactive nitrogen species. An imbalance between the formation of oxidative compounds and antioxidant defense mechanisms leads to oxidative damage [44]. ROS generated during inflammation directly damages proteins and lipids [49], thereby increasing oxidative stress [47]. Inflammation and oxidative stress influence the immune response with an induction of angiogenesis, tumor growth and metastasis [48, 49]. The experimental evidence of the antioxidative and anti-inflammatory properties of apoA-IV as shown for atherosclerosis in mice [13–15] may also explain the association of increased apoA-IV concentrations and lower number of cancer outcomes and cancer death in the GCKD study. Direct modulatory effects of apoA-IV on oxidation-induced intracellular redox-dependent cell signalling mechanisms were supported by cell culture studies [50].

Generalized endothelial dysfunction is evident in early stages of CKD, leads to albuminuria in the glomeruli and worsens with CKD progression [51]. Endothelial cells release proangiogenic factors which promote coagulation and inflammation [46]. The large endothelial surface of highly vascularized kidney vessels is particularly vulnerable to local proinflammatory effects. Endothelial activation attenuates the local vasodilatory capacity and thus increases the production of ROS and oxidized low-density- lipoprotein cholesterol [44, 48]. While healthy endothelial cells limit tumor growth, invasiveness, and metastasis, dysfunctional ones exposed to the inflammatory tumor microenvironment promote cancer progression, death, and even metastasis through NF-kappa B signaling and other cytokines [46, 48, 52, 53]. ApoA-IV was shown to inhibit NF-κB activity and upregulate the anti-oxidant and anti-apoptotic enzyme 24-Dehydrocholesterol reductase (DHCR24) [43]. Importantly, in vivo, already very low concentrations of apoA-IV could be considered anti-inflammatory [43]. This makes it interesting as a potential therapeutic target. Moreover, as the inhibition of vascular cell adhesion molecule 1 but not intercellular adhesion molecule 1 in vitro by lipid-bound apoA-IV was not fully dependent on DHCR24, other pathways besides inhibition of NF-κB might be of relevance for its anti-inflammatory effects [43]. ApoA-IV in its lipid-free and HDL-bound form was described to interact with scavenger receptor class B type 1 [54], which might be one possible alternate pathway. Lipid-bound apoA-IV might also increase the bioavailability of nitric oxide in endothelial cells via different pathways. Taken together, Shearston et al. showed that apoA-IV strongly inhibited acute vascular inflammation in vitro and in vivo [43]. This could be a further link to relevant mechanisms responsible for the lower number of cancer outcomes together with increased apoA-IV concentrations in the GCKD study.

A further explanation for our findings in the GCKD study is the growing evidence that HDL particles and their components including apoA-IV might either be involved in cancer development or might at least have diagnostic utility. A large meta-analysis [55] found an inverse relationship between plasma HDL-C levels and the risk of developing cancer. Cancer cells have an increased need of cholesterol and a disturbed cholesterol metabolism with an increase of intracellular cholesterol esters. This abnormal lipid and cholesterol metabolism in tumor cells can lead to altered plasma lipid levels in cancer patients. Disturbances in the cholesterol homeostasis of the cancer cells can therefore have an influence on progression of cancer as well as survival of cancer cells (reviewed in [55]).

In addition to lipid- and LDL-lowering effects, statins exhibit protective effects against DNA damage, improve endothelial function, and exert antioxidant and anti-inflammatory effects, and are associated with a reduced incidence for many cancers, which makes them potentially valuable in cancer prevention [48]. Importantly, the effect of higher apoA-IV concentration on decreased risk for cancer outcomes in the GCKD study was independent of statin use. Furthermore, in a study in hemodialysis patients we found no significant effect of atorvastatin on apoA-IV concentrations [22].

Regardless of the degree of renal dysfunction, CKD patients have a higher risk for venous thromboembolism compared to the general population [56]. Thrombosis-related death is the second most frequent cause in cancer patients [57]. A recent study has shown that apoA-IV reduced αIIbβ3 mediated-platelet aggregation and thus thrombosis. Aspirin or clopidogrel showed similar inhibitory effects as compared to apoA-IV [16]. As CKD and thrombosis, as well as cancer are linked, one mechanism how apoA-IV could be protective for cancer outcomes might be this recently demonstrated strong antithrombotic effect.

It is currently unclear whether apoA-IV concentrations measured in serum reflect the concentrations of apoA-IV in the microenvironment of the cancer and how this influences the survival of cancer cells. A recent study in patients with pancreatic ductal adenocarcinoma observed markedly decreased apoA-IV concentrations in plasma but a pronounced increase of mRNA in the diseased pancreatic tissue which was linked with a less favourable outcome of the patients. This was surprising since usually RNA expression of apoA-IV in normal pancreas tissue is almost zero [58]. Decreased apoA-IV concentrations in pancreatic cancer were recently confirmed by a study in cats [59].

### Strengths and limitations of the study

The main advantage of the current study compared to most of the proteomic studies is the use of a validated ELISA for measurement of apoA-IV and the prospective assessment of cancer outcomes. Particular strengths of the GCKD study are the large sample size based on a well-defined population with a median follow-up of 6.5 years with very low loss to follow-up, the homogeneity of the study population and a centralized assessment of clinical outcomes.

Since the GCKD study is designed as an observational epidemiological study, it cannot determine causality or conclude on possible biological mechanisms. In case the association of apoA-IV with outcomes is not causal it can at least be considered as a promising predictor of risk for cancer. However, the experimental data from earlier studies are in support of a causal involvement of apoA-IV in outcomes involved in inflammatory processes [14–16, 60, 61]. A further limitation is that we do not have a replication cohort available and that mainly CKD patients in stage G3 or A3 are included in the GCKD study and therefore findings might not be generalizable to other stages of CKD or the general population. However, previous proteomic studies mostly done in serum or plasma revealed that apoA-IV is reduced in many cancer types (for overview see Supplementary Table 1). Furthermore, we cannot exclude residual confounding by unknown or unmeasured confounders. Finally, apoA-IV was measured in serum and no organ-specific cancer tissue was available which would have allowed to analyse the apoA-IV expression in cancer tissue.

## Conclusions

This large prospective study in patients with mild to severe chronic kidney disease revealed an independent association of high apoA-IV concentrations with a lower odds for a history of cancer before enrolment and a lower risk for fatal and non-fatal incident cancer outcomes. The known antioxidative, antiinflammatory and potentially also antithrombotic properties of apoA-IV might be an explanation for the observed associations. Supported by existing proteomic data this could mean a value of high apoA-IV indicating a reduced risk for cancer in CKD patients. However, the clinical utility requires further investigations of various cancer entities in large cohorts to define optimal thresholds for further clinical and extended examinations of patients in case a cancer is suspected due to decreased apoA-IV concentrations.

### Supplementary Information

**Additional file 1.** The online supplementary material (Supplementary Tables_ApoA-IV_CA.pdf) contains a literature overview on proteomics and case –control studies that investigated apolipoprotein A-IV in various cancer types (Supplementary Table 1), distribution of type of cancer during the prospective observation period (Supplementary Table 2) further baseline characteristics of the GCKD cohort (Supplementary Table 3) as well as additional data analyses on the association of apolipoprotein A-IV with cancer outcomes (Supplementary Table 4-13).

## Data Availability

The data that support the findings of this study are available from the corresponding author upon reasonable request.

## Abbreviations

ApoA-IV: Apolipoprotein A-IV
CKD: Chronic kidney disease
DHCR24: 24-Dehydrocholesterol reductase
eGFR: Estimated glomerular filtration rate
eGFRcys: EGFR based on cystatin C
eGFRcrea: EGFR based on creatinine
GCKD Study: German Chronic Kidney Disease Study
HR: Hazard ratio
hs-CRP: High sensitivity C reactive protein
ROS: Reactive oxygen species
UACR: Urine albumin creatinine ratio
