# Correction: Apolipoprotein A-IV concentrations and cancer in a large cohort of chronic kidney disease patients: results from the GCKD study

**DOI:** 10.1186/s12885-024-12128-6

**Published:** 2024-03-19

**Authors:** Barbara Kollerits, Simon Gruber, Inga Steinbrenner, Johannes P. Schwaiger, Hansi Weissensteiner, Sebastian Schönherr, Lukas Forer, Fruzsina Kotsis, Ulla T. Schultheiss, Heike Meiselbach, Christoph Wanner, Kai-Uwe Eckardt, Florian Kronenberg, for the GCKD Investigators

**Affiliations:** 1 Institute of Genetic Epidemiology, Medical University of Innsbruck, Schöpfstraße 41, 6020 Innsbruck, Austria; 2 Institute of Genetic Epidemiology, Faculty of Medicine and Medical Center - University of Freiburg, Freiburg, Germany; 3 Department of Nephrology and Hypertension, University Hospital Erlangen, Friedrich-Alexander-Universität Erlangen-Nürnberg, Erlangen, Germany; 4 German Chronic Kidney Disease Study, Erlangen, Germany; 5 Department of Medicine IV – Nephrology and Primary Care, Faculty of Medicine and Medical Center - , University of Freiburg, Freiburg, Germany; 6 Division of Nephrology, Department of Internal Medicine I, University Hospital Würzburg, Würzburg, Germany; 7 Department of Nephrology and Medical Intensive Care, Charité – Universitätsmedizin Berlin, Berlin, Germany

**Correction** **:** **BMC Cancer 24, 320 (2024)**

10.1186/s12885-024-12053-8

Following publication of the original article [1], the author noticed an error in the affiliation of Inga Steinbrenner. Her affiliation should be 2, instead of affiliation 3.

The correct affiliation for Inga Steinbrenner is:

^2^Institute of Genetic Epidemiology, Faculty of Medicine and Medical Center—University of Freiburg, Freiburg, Germany.

The original article [1] has been corrected.
