# Supplementary material for: Apolipoprotein A-IV concentrations and cancer in a large cohort of chronic kidney disease patients: results from the GCKD study
Source: BMC Cancer. 2024 Mar 7;24:320. doi: 10.1186/s12885-024-12053-8 (PMC10921727; doi:10.1186/s12885-024-12053-8)
Supplement: Supplementary file 1 — Additional file 1. The online supplementary material (Supplementary Tables_ApoA-IV_CA.pdf) contains a literature overview on proteomics and case –control studies that investigated apolipoprotein A-IV in various cancer types (Supplementary Table 1), distribution of type of cancer during the prospective observation period (Supplementary Table 2) further baseline characteristics of the GCKD cohort (Supplementary Table 3) as well as additional data analyses on the association of apolipoprotein A-IV with cancer outcomes (Supplementary Table 4-13). [file 12885_2024_12053_MOESM1_ESM.pdf]

Supplementary Tables to

Apolipoprotein A-IV concentrations and cancer in a large cohort of chronic kidney disease patients: results from the GCKD study

Barbara Kollerits [1], Simon Gruber [1], Inga Steinbrenner [3], Johannes P. Schwaiger [1], Hansi Weissensteiner [1], Sebastian Schönherr [1], Lukas Forer [1], Fruzsina Kotsis [2,4], Ulla T. Schultheiss [2,4], Heike Meiselbach [3], Christoph Wanner [5], Kai-Uwe Eckardt [3,6], Florian Kronenberg [1]; for the GCKD Investigators

[1] *Institute of Genetic Epidemiology, Medical University of Innsbruck, Innsbruck, Austria*

[2] *Institute of Genetic Epidemiology, Faculty of Medicine and Medical Center - University of Freiburg, Freiburg, Germany*

[3] *Department of Nephrology and Hypertension, University Hospital Erlangen, Friedrich-Alexander-Universität Erlangen-Nürnberg, Erlangen, Germany and German Chronic Kidney Disease study*

[4] *Department of Medicine IV – Nephrology and Primary Care, Faculty of Medicine and Medical Center - University of Freiburg, Freiburg, Germany*

[5] *Division of Nephrology, Department of Internal Medicine I, University Hospital Würzburg, Würzburg, Germany*

[6] *Department of Nephrology and Medical Intensive Care, Charité – Universitätsmedizin Berlin, Berlin, Germany*

Note: The results presented in this manuscript were part of the diploma thesis of Simon Gruber for graduation at the Medical School of the Medical University of Innsbruck.

Corresponding author:

Florian Kronenberg, MD
Institute of Genetic Epidemiology
Medical University of Innsbruck
Schöpfstraße 41, A-6020 Innsbruck, Austria.
Phone: (+43) 512-9003-70560
Fax: (+43) 512 9003-73560 or -73561,
Email: Florian.Kronenberg@i-med.ac.at

Supplementary Table 1: Biomarker studies that investigated apolipoprotein A-IV in various cancer types: **Green letters** in case expression or concentration of apoA-IV is increased, **red letters** in case expression or concentration of apoA-IV is decreased. Yellow highlighted are results of studies in which measurement or validation was performed by a quantitative apoA-IV assay.

Study	Design and recruitment	Patient numbers	Material	Method	Main results
**Ovarian cancer**					
Dieplinger et al. 2009 *Cancer Epidem Biom Prev* [1]	Cases – benign gynaecologic conditions – controls	**181** - 399 - 177	Plasma	**ELISA**	ApoA-IV **reduced** in cases vs. benign gynaecologic conditions vs. controls: 9.4 mg/dl vs. 11.7 mg/dl vs. 13mg/dl; No independent diagnostic information of A-IV to CA125 and age for differentiation of the disease status
Lorkova et al. 2012 *Oncology Reports* [2]	Cases – controls Before surgery and chemotherapy	10 - 10	Serum	1) Proteomic analysis 2) Validation **ELISA**	ApoA-IV **decreased** in 10 cases versus 10 controls (roughly 15 mg/dL vs. 48 mg/dL, estimated from Figure 2 of the publication)
Li et al. 2012 Asian Pac J Cancer Prev [3]	Cancer – benign tumors – controls	21 - 16 - 20	Serum	1) Proteomic analysis 2) Validation by Western blot	**Decreased** apoA-IV expression
Timms et al. 2014 *Proteomics Clin Appl* [4]	A) Cases vs. benign ovarian conditions vs. controls B) Validation by ELISA: malignant vs. benign	A) 22 – 45 – 64 B) 22 - 45 from A) and 48 - 22 independent samples	Serum	A) Proteomic analysis B) Validation in additional samples by **ELISA**	**Lower** apoA-IV significantly discriminated benign from malignant but not as good as CA125
Rauniyar et al. 2017 *Biomarker Insights* [5]	Cases – controls	6 – 7	Serum	Proteomic analysis	ApoA-IV significantly **decreased**
**Cervical cancer**					
Jeong et al. 2008 *J Gynecol Oncol* [6]	Cases – controls	6 - 6	Plasma	Proteomic analysis	ApoA-IV precursor **downregulated**
**Endometrial cancer**					
Wang et al. 2011 *J Haem & Onc* [7]	Endometrial lesions various stages - carcinoma - healthy controls	14 - 6 - 7	Serum	Proteomic analysis	Downregulation of A-IV precursor in complex and atypical endometrial hyperplasia, but not in endometrial carcinoma
**Hepatocellular cancer**					
Kawakami et al. 2005 *Proteomics* [8]	Before and after radiofrequency ablation treatment	8	Serum	Proteomic analysis	ApoA-IV precursor **decreased** after treatment
Pleguezuelo et al. 2010 *World J Hepat* [9]	Cases versus liver cirrhosis	18 - 22	Plasma	Proteomic analysis	ApoA-IV significantly **higher** in patients with hepatocellular carcinoma compared to liver cirrhosis
Sugimoto et al. 2013 *Int J of Mol Med* [10]	Hepatitis-C-induced cirrhosis - cirrhosis -hepatocellular carcinoma - controls	24 – 17 - 19 - 19	Serum	Proteomic analysis	**Reduced** level of apoA-IV isoform in liver cirrhosis and hepatocellular carcinoma
Bharali et al. 2018 *Indian J Med Res* [11]	Cases - liver cirrhosis - chronic hepatitis - healthy controls	50 – 25 – 25 - 10	Plasma	1) Western Blot 2) Validation by **ELISA**	**Decreased** apoAIV concentrations in hepatocellular carcinoma compared to liver cirrhosis and chronic hepatitis
**Extrahepatic cholangio-carcinoma (CCA)**					
Son et al. 2020 *J Cancer 2020* [12]	CCA - benign biliary conditions	18 - 5	Bile	Proteomic analysis	Non-significant increase of apoA-IV expression in **bile** of CCA patients
**Pancreatic cancer**					
Abulaizi et al. 2011 *Int J Proteomics* [13]	Cases - controls	32 – 32	Serum	1) Proteomic analysis 2) Validation by **ELISA**	ApoA-IV significantly **decreased** in 15 cases versus 15 controls: 107.8 ± 25.8 vs. 185.3 ± 16.0 arbitrary units
Park et al. 2017 *Oncotarget* [14]	Cases - pancreatitis - controls	Discovery: 116 - 31 - 35 Validation: 292 - 70 - 94	Serum	Discovery: proteomic and gene expression Validation: proteomic analysis	Significantly **decreased** apoA-IV expression in cancer cases
Peng et al. 2020 *Cancers* [15]	Cases – controls	Pilot cohort 10 - 10 Testing cohort: 50 - 49	Plasma	Proteomic analysis RNA sequencing	ApoA-IV significantly **decreased** compared to controls

Study	Design and recruitment	Patient numbers	Material	Method	Main results
**Gastric cancer**					
Liu et al. 2012 *Clin Chim Acta* [16]	Cases - controls	20 - 10	Serum	Proteomic analysis	ApoA-IV precursor **upregulation**
**Colorectal cancer**					
Sugimachi et al. 2016 *Ann Surg Oncol* [17]	Consecutive primary CRC patients undergoing surgery	107	cDNA	ApoA-IV gene expression levels	**Higher mRNA apo A-IV expression** was associated with a poor prognosis
Ahn et al. 2019 *Clin Proteom* [18]	Cases (different stages) - controls	80 - 20	Plasma	Proteomic analysis	**Decreased** apo A-IV expression in cases
**Colorectal and prostate cancer**					
Karczmarski et al. 2013 *Acta biochem Pol* [19]	Cancer cases (16 colorectal and 28 prostate cancer) - controls	44 - 86	Serum	Mass spectrometry of proteolytic fragments	Two APOA4 peptides were found to be **higher** in colorectal and prostate cancer compared to controls
**Oral cancer**					
Chang et al. 2019 *J Food and Drug Anal* [20]	Cases - controls	40 cases in test group and 71 cases in validation vs. 55 controls	Plasma	1) Proteomic analysis 2) Validation by **ELISA**	apo A-IV and apo A-IV/total protein ratios were significantly **decreased** in plasma of patients versus controls
**Thyroid cancer**					
Abdullah et al. 2016 *PeerJ* [21]	Cases with and without history of benign thyoid goitre - controls	6 - 8 - 20	Tissue and serum	Proteomic analysis	**Enhanced apoA-IV expression** in papillary thyroid cancer patients with history of benign thyroid goitre
Farrokhi Yekta et al. 2018 Int J Biol Mark [22]	Cases - multinodal goitre - controls Newly diagnosed patients	17 - 17 - 20	Serum	1) Proteomic analysis 2) Validation by **ELISA**	**Decreased** apoA-IV in papillary thyroid cancer
Li et al. 2020 *PeerJ 2020* [23]	Cases - benign nodules - controls	Training: 29 - 15 - 10 Validation: 44 - 20 - 15	Serum	1) Proteomic analysis 2) Validation by **ELISA**	ApoA-IV strongly **downregulated** in cancer cases but **higher** concentrations in the validation study
**Lung cancer**					
Dowling et al. 2007 *Electrophoresis* [24]	6 cases versus pooled standard of 16 controls	6 versus pooled standard	Serum	Proteomic analysis	**Increased** abundance of apoAIV-precursor in cases
Okano et al. 2016 Int J Canc [25]	Cases - COPD - healthy smokers Nippon Medical School, Japan	11 – 7 - 7	Serum	1) Proteomic analysis 2) Western Blot in subsample	**Increased** apoA-IV expression in lung cancer cases
**Acute myeloid leukaemia**					
Zheng et al. 2017 *Gen & Mol Res* [26]	Cases - controls	62 - 15	Serum	Proteomic analysis	**Decreased** apoA-IV expression
**Glioblastoma**					
Miyauchi et al. 2018 *PLOS One* [27]	Cases - controls	14 - 15	Plasma	Proteomic analysis	ApoA-IV expression significantly **decreased**
**Bladder cancer**					
Soukup et al. 2019 *Neoplasma [28]*	Cases - controls	90 - 60	**Urine**	**ELISA**	Significantly **higher urinary apoA-IV values** in cases vs. controls

Supplementary Table 2: Type of cancer observed during the prospective observation period. Only the first event is counted.

**Type of cancer**	**Number (n=368)**
Renal tract cancers (kidney, bladder, urothelial cancer)	61
Male cancers (prostate cancer, others)	55
Digestive system (oesophagus, stomach, small intestine, colon, rectum)	47
Skin cancer (melanoma, squamous cell carcinoma)	49
Female cancers (breast, cervix, uterus, ovary)	41
Lung cancer	40
Haematological cancers (lymphoma, leukaemia, multiple myeloma, and other malignant haematological conditions)	33
Abdominal solid organs (liver, gallbladder, biliary tract, pancreas, other digestive organs)	23
Other cancers	15
Unknown cancer	4

Supplementary Table 3: Baseline characteristics of German Chronic Kidney Disease (GCKD) study patients stratified by quartiles of apolipoprotein A-IV

	Apolipoprotein A-IV quartiles				
	Quartile 1 (n=1257)	Quartile 2 (n=1253)	Quartile 3 (n=1262)	Quartile 4 (n=1267)	p-value for trend
ApoA-IV (mg/dL): range	5.2-22.0	22.0-27.6	27.6-34.0	34.0-100.2	
Mean±SD	18.1±3.0	24.8±1.6	30.6±1.8	42.0±7.6	-
25th, 50th and 75th percentile	[16.2;18.6;20.5]	[23.4;24.8;26.1]	[29.0;30.4;32.0]	[36.5;39.8;45.2]	
Age (years)	60±12 [52;63;70]	61±12 [55;64;70]	61±12 [55;64;70]	59±12 [51;62;69]	0.007
Female gender, n (%)	573 (46)	487 (39)	476 (38)	469 (37)	<0.001
Body mass index, (kg/m^2)	30.3±6.1 [26.2;29.7;33.6]	30.0±6.0 [25.8;29.1;33.5]	29.9±6.0 [25.9;28.8;33;1]	29.0±5.6 [25.0;28.2;32.4]	<0.001
Smoker and ex-smoker, n (%)	702 (56)	707 (57)	759 (60)	798 (63)	<0.001
Diabetes, n (%)	374 (30)	430 (34)	467 (37)	515 (41)	<0.001
Hypertension, n (%)	1168 (93)	1210 (97)	1218 (97)	1253 (99)	<0.001
Cardiovascular disease, n (%)	326 (26)	312 (25)	353 (28)	296 (23)	0.35
eGFR Cystatin C (mL/min/1.73m^2)	60±22 [45;56;72]	52±19 [39;50;62]	47±18 [35;44;56]	40±15 [29;38;49]	<0.001
eGFR Creatinine (mL/min/1.73m^2)	58±20 [44;54;67]	51±17 [40; 49; 59]	47±17 [36; 44; 55]	42±14 [31; 40; 48]	<0.001
UACR (mg/g)	199±586 [6;19;106]	274±714 [7;31;193]	429±974 [11;55;383]	815±1285 [39;281;1078]	<0.001
Statin use, n (%)	538 (43)	553 (44)	619 (49)	685 (54)	<0.001
Serum albumin,(g/L)	39.0±4.0 [36.9;39.3;41.3]	38.9±4.0 [36.9;39.1;41.2]	38.3±4.6 [36.3;38.5;40.6]	37.3±4.8 [35.2;37.8;40.3]	<0.001
Hemoglobin, (g/dL)	13.9±1.7 [12.8;13.9;15.0]	13.8±1.6 [12.8;13.8;14.9]	13.6±1.6 [12.5;13.5;14.7]	13.2±1.7 [12.1;13.1;14.3]	<0.001
Hs-CRP, (mg/L)	6.7±12.7 [1.3;2.9;7.0]	4.7±6.9 [1.1;2.3;5.0]	4.2±6.3 [1.0;2.2;4.6]	3.4±4.7 [0.8;1.9;3.9]	<0.001
Total cholesterol, (mg/dL)	204±47 [171;203;231]	211±50 [177;208;240]	210±50 [174;207;238]	221±61 [181;213;251]	<0.001
LDL cholesterol, (mg/dL)	116±39 [88;112;141]	119±43 [91;115;144]	117±42 [89;113;141]	122±50 [89;114;148]	0.15
HDL cholesterol, (mg/dL)	50±16 [38;47;58]	51±17 [39;47;59]	51±18 [39;48;61]	56±21[42;52;67]	<0.001
Triglycerides, (mg/dL)	183±104 [113;160;224]	200±120 [123;169;240]	205±139[120;175;249]	209±143 [117;171;256]	<0.001

Values are provided as mean ± standard deviation and [25th; 50th (median); and 75th percentiles] or as number of patients (%). % (=percentage considering missing values). In total group, for all variables displayed, number of missing values ≤2.5% (n=5039). eGFR calculated according to the CKD-EPI equation [29]. Hs-CRP and urine-albumin values that were below the lower detection limit (LOD) were replaced by LOD/√2. BMI was corrected for amputation. UACR calculated according to the following equation: Albumin in urine (mg/l) x 100 / Creatinine in urine (mg/dl) and is given in mg/g. Hypertension was defined as systolic blood pressure ≥140 mmHg and/or diastolic blood pressure ≥90 mmHg, and/or receiving antihypertensive treatment. Cardiovascular disease was defined as myocardial infarction, coronary artery bypass grafting, percutaneous transluminal coronary angioplasty, stroke, interventions at the carotid arteries.

Supplementary Table 4: Association of apolipoprotein A-IV with history of cancer at the baseline investigation. Data are as in model 2 from Table 2 but additionally adjusted for HDL-C, LDL-C and triglycerides.

History of cancer		OR	95% CI	p-value
**Calculations for median of apoA-IV concentrations** [a]				
Model 2: 601 cases (269 above and 332 below median) [b]		0.81	0.67-0.98	0.03
**Calculations per quartile of ApoA-IV concentrations**				
Model 2	Quartile 1 (163 cases) [b]	1.00		
	Quartile 2 (169 cases) [b]	1.01	0.79-1.28	0.95
	Quartile 3 (142 cases) [b]	0.82	0.64-1.06	0.13
	Quartile 4 (127 cases) [b]	0.80	0.60-1.07	0.13

[a] Reference category includes apoA-IV values below median. The median apoA-IV concentration is 27.6 mg/dL. (referring to the total group of 5039 patients).

[b] "Cases" refers to the number of patients with a history of cancer.

Data adjusted for age, sex, $eGFR_{creatinine}$, ln-urine albumin-creatinine ratio, statin use, smoking, BMI, diabetes, HDL-C, LDL-C, and triglycerides.

Abbreviations: OR, odds ratio; CI, confidence interval

Supplementary Table 5: Association of apolipoprotein A-IV with incident cancer without a history of cancer at the baseline investigation [a]. Data are as in model 2 from Table 3 but additionally adjusted for HDL-C, LDL-C and triglycerides.

**Incident cancer**		**HR**	**95% CI**	**p-value**
**Calculations for median of apoA-IV concentrations [b]**				
Model 2: 360 cases (171 above and 189 below median) [c]		0.73	0.58-0.92	0.007
**Calculations per quartile of ApoA-IV concentrations**				
Model 2	Quartile 1 (88 cases) [c]	1.00		
	Quartile 2 (101 cases) [c]	1.01	0.76-1.35	0.94
	Quartile 3 (83 cases) [c]	0.73	0.53-1.00	0.05
	Quartile 4 (88 cases) [c]	0.74	0.53-1.04	0.08

[a] Patients with a history of cancer at the time of enrollment were not considered in this analysis.

[b] Reference category includes apoA-IV values below median. The median apoA-IV concentration is 27.6 mg/dL (referring to the total group of 5039 patients).

[c] "Cases" refers to the number of patients with incident cancer events.

Data adjusted for age, sex, $eGFR_{cystatin\text{-}C}$, ln-urine albumin-creatinine ratio, statin use, smoking, BMI, diabetes, HDL-C, LDL-C, and triglycerides.

Abbreviations: HR, hazard ratio; CI, confidence interval

Supplementary Table 6: Association of apolipoprotein A-IV with fatal cancer without a history of cancer at the baseline investigation [a]. Data are as in model 2 from Table 4 but additionally adjusted for HDL-C, LDL-C and triglycerides.

Fatal cancer		HR	95% CI	p-value
**Calculations per 10 mg/dL increment of apoA-IV concentrations**				
Model 2: 59 cases [b]		0.62	0.44-0.88	0.007
**Calculations per quartile of ApoA-IV concentrations**				
Model 2	Quartile 1 (19 cases) [b]	1.00		
	Quartile 2 (10 cases) [b]	0.43	0.20-0.92	0.03
	Quartile 3 (19 cases) [b]	0.66	0.34-1.28	0.22
	Quartile 4 (11 cases) [b]	0.34	0.15-0.78	0.01

[a] Patients with a history of cancer at the time of enrollment were not considered in this analysis.

[b] "Cases" refers to the number of patients with fatal cancer events.

Data adjusted for age, sex, eGFR $_{cystatin\text{-}C}$, ln-urine albumin-creatinine ratio, statin use, smoking, BMI, diabetes, HDL-C, LDL-C, and triglycerides.

Abbreviations: HR, hazard ratio; CI, confidence interval

Supplementary Table 7: Association of apolipoprotein A-IV with incident cancer during the prospective follow-up. Analysis includes also patients who already had a history of cancer at the baseline investigation.

Incident cancer		HR	95% CI	p-value
**Calculations for median of apoA-IV concentrations** [a]				
Model 1: 445 cases (206 above and 239 below median) [b]		0.73	0.60-0.90	0.003
Model 2: 438 cases (202 above and 136 below median) [b]		0.73	0.59-0.89	0.003
**Calculations per quartile of ApoA-IV concentrations**				
Model 1	Quartile 1 (107 cases) [b]	1.00		
	Quartile 2 (132 cases) [b]	1.06	0.82-1.38	0.64
	Quartile 3 (105 cases) [b]	0.78	0.59-1.03	0.08
	Quartile 4 (101 cases) [b]	0.74	0.55-1.00	0.05
Model 2	Quartile 1 (105 cases) [b]	1.00		
	Quartile 2 (131 cases) [b]	1.08	0.83-1.39	0.58
	Quartile 3 (102 cases) [b]	0.77	0.58-1.03	0.07
	Quartile 4 (100 cases) [b]	0.74	0.55-1.01	0.06

[a] Reference category includes apoA-IV values below median The median apoA-IV concentration is 27.6 mg/dL (referring to the total group of 5039 patients)

[b] "Cases" refers to the number of patients with incident cancer events. Differences in number of cases between model 1 and 2 are explained by few patients with some missing covariates for model 2.

Model 1: adjusted for age, sex, $eGFR_{cystatin\text{-}C}$, ln-urine albumin-creatinine ratio

Model 2: as model 1 plus statin use, smoking, BMI, diabetes, history of cancer

Abbreviations: HR, hazard ratio; CI, confidence interval

Supplementary Table 8: Association of apolipoprotein A-IV with fatal cancer during the prospective follow-up. Analysis includes also patients who already had a history of cancer at the baseline investigation.

**Fatal cancer** [a]		**HR**	**95% CI**	**p-value**
**Calculations per 10 mg/dL increment of apoA-IV concentrations**				
Model 1: 95 cases [a]		0.72	0.55-0.95	0.02
Model 2: 92 cases [a]		0.74	0.57-0.98	0.04
**Calculations per quartile of apoA-IV concentrations**				
Model 1	Quartile 1 (31 cases) [a]	1.00		
	Quartile 2 (16 cases) [a]	0.43	0.24-0.79	0.007
	Quartile 3 (28 cases) [a]	0.69	0.41-1.18	0.18
	Quartile 4 (20 cases) [a]	0.50	0.27-0.93	0.03
Model 2	Quartile 1 (29 cases) [a]	1.00		
	Quartile 2 (16 cases) [a]	0.47	0.26-0.87	0.02
	Quartile 3 (27 cases) [a]	0.75	0.43-1.29	0.30
	Quartile 4 (20 cases) [a]	0.55	0.29-1.05	0.07

[a] "Cases" refers to the number of patients with fatal cancer events. Differences in number of cases between model 1 and 2 are explained by few patients with some missing covariates for model 2.

Model 1: adjusted for age, sex, $eGFR_{cystatin-C}$, ln-urine albumin-creatinine ratio

Model 2: as model 1 plus statin use, smoking, BMI, diabetes, history of cancer

Abbreviations: HR, hazard ratio; CI, confidence interval

Supplementary Table 9: Association of apolipoprotein A-IV with history of cancer. Data are as in model 2 from Table 2 but additionally adjusted for hs-CRP.

	OR	95% CI	p-value
**Calculations for median of apoA-IV concentrations** [a]			
Model 2: 604 cases (270 above and 334 below median) [b]	0.85	0.71-1.04	0.11
Model 2: 603 cases (269 above and 334 below median) [b]	0.81[¥]	0.67-0.98	0.03
**Calculations per quartile of ApoA-IV concentrations**			
Model 2			
Quartile 1 (163 cases) [b]	1.00		
Quartile 2 (171 cases) [b]	1.06	0.84-1.35	0.63
Quartile 3 (142 cases) [b]	0.88	0.68-1.13	0.32
Quartile 4 (128 cases) [b]	0.89	0.67-1.19	0.43
Model 2			
Quartile 1 (163 cases) [b]	1.00		
Quartile 2 (171 cases) [b]	1.03[c]	0.81-1.30	0.83
Quartile 3 (142 cases) [b]	0.83 [c]	0.64-1.07	0.16
Quartile 4 (127 cases) [b]	0.81 [c]	0.61-1.07	0.14

[a] Reference category includes apoA-IV values below median. The median apoA-IV concentration is 27.6 mg/dL (referring to the total group of 5039 patients). Differences in number of cases between the two models are explained by few patients with some missing covariates for model 2 adjusted for $GFR_{creatinine}$.

[b] "Cases" refers to the number of patients with a history of cancer.

Data adjusted for age, sex, $eGFR_{cystatin-C}$, ln-urine albumin-creatinine ratio, statin use, smoking, BMI, diabetes and ln-hs-CRP

[c] adjusted for $eGFR_{creatinine}$ instead of $eGFR_{cystatin-C}$

Abbreviations: OR, odds ratio; CI, confidence interval

Supplementary Table 10: Association of apolipoprotein A-IV with incident cancer without a history of cancer at the baseline investigation [a]. Data are as in model 2 from Table 3 but additionally adjusted for hs-CRP.

**Incident cancer**		**HR**	**95% CI**	**p-value**
**Calculations for median of apoA-IV concentrations [b]**				
Model 2: 362 cases (171 above and 191 below median) [c]		0.74	0.59-0.93	0.01
**Calculations per quartile of ApoA-IV concentrations**				
Model 2	Quartile 1 (88 cases) [c]	1.00		
	Quartile 2 (103 cases) [c]	1.05	0.78-1.40	0.77
	Quartile 3 (83 cases) [c]	0.75	0.55-1.03	0.07
	Quartile 4 (88 cases) [c]	0.78	0.57-1.10	0.15

[a] Patients with a history of cancer at the time of enrollment were not considered in this analysis.

[b] Reference category includes apoA-IV values below median. The median apoA-IV concentration is 27.6 mg/dL (referring to the total group of 5039 patients).

[c] "Cases" refers to the number of patients with fatal cancer events.

Data adjusted for age, sex, eGFR $_{cystatin\text{-}C}$, ln-urine albumin-creatinine ratio, statin use, smoking, BMI, diabetes, and ln-hs-CRP

Abbreviations: HR, hazard ratio; CI, confidence interval

Supplementary Table 11: Association of apolipoprotein A-IV with fatal cancer without a history of cancer at the baseline investigation [a]. Data are as in model 2 from Table 4 but additionally adjusted for hs-CRP.

Fatal cancer		HR	95% CI	p-value
**Calculations per 10 mg/dL increment of apoA-IV concentrations**				
Model 2: 60 cases [b]		0.65	0.45-0.92	0.02
**Calculations per quartile of ApoA-IV concentrations**				
Model 2	Quartile 1 (19 cases) [b]	1.00		
	Quartile 2 (11 cases) [b]	0.48	0.23-1.01	0.05
	Quartile 3 (19 cases) [b]	0.69	0.36-1.35	0.28
	Quartile 4 (11 cases) [b]	0.38	0.17-0.88	0.02

[a] Patients with a history of cancer at the time of enrollment were not considered in this analysis.

[b] "Cases" refers to the number of patients with fatal cancer events.

Data adjusted for age, sex, eGFR $_{cystatin\text{-}C}$, ln-urine albumin-creatinine ratio, statin use, smoking, BMI, diabetes, and ln-hs-CRP

Abbreviations: HR, hazard ratio; CI, confidence interval

Supplementary Table 12: Association of apolipoprotein A-IV with incident cancer during the prospective follow-up without a history of cancer at the baseline investigation based on subdistribution hazard ratio (SHR) models (4424 out of 5039 patients) [a]

Incident cancer		SHR	95% CI	p-value
**Calculations for median of apoA-IV concentrations** [b]				
Model 1: 368 cases(174 above and 194 below median) [c]		0.76	0.61-0.95	0.02
Model 2: 362 cases(174 above and 194 below median) [c]		0.77	0.62-0.96	0.02
**Calculations per quartile of ApoA-IV concentrations**				
Model 1	Quartile 1 (90 cases) [c]	1.00		
	Quartile 2 (104 cases) [c]	1.04	0.78-1.38	0.80
	Quartile 3 (85 cases) [c]	0.77	0.56-1.04	0.09
	Quartile 4 (89 cases) [c]	0.79	0.57-1.10	0.16
Model 2	Quartile 1 (88 cases) [c]	1.00		
	Quartile 2 (103 cases) [c]	1.05	0.79-1.40	0.73
	Quartile 3 (83 cases) [c]	0.76	0.56-1.04	0.08
	Quartile 4 (88 cases) [c]	0.79	0.57-1.11	0.18

[a] Patients with a history of cancer at the time of enrollment were not considered in this analysis.

[b] Reference category includes apoA-IV values below median. The median apoA-IV concentration is 27.6 mg/dL (referring to the total group of 5039 patients).

[c] "Cases" refers to the number of patients with incident cancer events. Differences in number of cases between model 1 and 2 are explained by patients with few missing covariates for model 2.

Model 1: adjusted for age, sex, $eGFR_{cystatin\text{-}C}$, ln-urine albumin-creatinine ratio
Model 2: as model 1 plus statin use, smoking, BMI, and diabetes

Abbreviations: SHR, subdistribution hazard ratio; CI, confidence interval

Supplementary Table 13: Association of apolipoprotein A-IV with fatal cancer during the prospective follow-up without a history of cancer at the baseline investigation based on subdistribution hazard ratio (SHR) models (4424 out of 5039 patients) [a]

**Fatal cancer**		**SHR**	**95% CI**	**p-value**
**Calculations per 10 mg/dL increment of apoA-IV concentrations**				
Model 1: 62 cases [b]		0.63	0.45-0.89	0.009
Model 2: 60 cases [b]		0.64	0.47-0.88	0.006
**Calculations per quartile of ApoA-IV concentrations**				
Model 1	Quartile 1 (21 cases) [b]	1.00		
	Quartile 2 (11 cases) [b]	0.44	0.21-0.91	0.03
	Quartile 3 (19 cases) [b]	0.65	0.34-1.23	0.18
	Quartile 4 (11 cases) [b]	0.35	0.15-0.81	0.01
Model 2	Quartile 1 (19 cases) [b]	1.00		
	Quartile 2 (11 cases) [b]	0.49	0.23-1.03	0.06
	Quartile 3 (19 cases) [b]	0.71	0.37-1.38	0.31
	Quartile 4 (11 cases) [b]	0.39	0.17-0.90	0.03

[a] Patients with a history of cancer at the time of enrollment were not considered in this analysis.
[b]"Cases" refers to the number of patients with fatal cancer events. Differences in number of cases between model 1 and 2 are explained by few patients with some missing covariates for model 2.Model 1: adjusted for age, sex, $eGFR_{cystatin-C}$, ln-urine albumin-creatinine ratio

Model 2: as model 1 plus statin use, smoking, BMI, and diabetes

Abbreviations: SHR, subdistribution hazard ratio; CI, confidence interval

References

1. Dieplinger H, Ankerst DP, Burges A, Lenhard M, Lingenhel A, Fineder L, Buchner H, Stieber P: **Afamin and apolipoprotein A-IV: novel protein markers for ovarian cancer**. *Cancer Epidemiol Biomarkers Prev* 2009, **18**:1127-1133.
2. Lorkova L, Pospisilova J, Lacheta J, Leahomschi S, Zivny J, Cibula D, Zivny J, Petrak J: **Decreased concentrations of retinol-binding protein 4 in sera of epithelial ovarian cancer patients: a potential biomarker identified by proteomics**. *Oncol Rep* 2012, **27**(2):318-324.
3. Li L, Xu Y, Yu CX: **Proteomic analysis of serum of women with elevated Ca-125 to differentiate malignant from benign ovarian tumors**. *Asian Pac J Cancer Prev* 2012, **13**(7):3265-3270.
4. Timms JF, Arslan-Low E, Kabir M, Worthington J, Camuzeaux S, Sinclair J, Szaub J, Afrough B, Podust VN, Fourkala EO *et al*: **Discovery of serum biomarkers of ovarian cancer using complementary proteomic profiling strategies**. *Proteomics Clin Appl* 2014, **8**:982-993.
5. Rauniyar N, Peng G, Lam TT, Zhao H, Mor G, Williams KR: **Data-Independent Acquisition and Parallel Reaction Monitoring Mass Spectrometry Identification of Serum Biomarkers for Ovarian Cancer**. *Biomark Insights* 2017, **12**:1177271917710948.
6. Jeong DH, Kim HK, Prince AE, Lee DS, Kim YN, Han J, Kim KT: **Plasma proteomic analysis of patients with squamous cell carcinoma of the uterine cervix**. *J Gynecol Oncol* 2008, **19**(3):173-180.
7. Wang YS, Cao R, Jin H, Huang YP, Zhang XY, Cong Q, He YF, Xu CJ: **Altered protein expression in serum from endometrial hyperplasia and carcinoma patients**. *J Hematol Oncol* 2011, **4**:15.
8. Kawakami T, Hoshida Y, Kanai F, Tanaka Y, Tateishi K, Ikenoue T, Obi S, Sato S, Teratani T, Shiina S *et al*: **Proteomic analysis of sera from hepatocellular carcinoma patients after radiofrequency ablation treatment**. *Proteomics* 2005, **5**:4287-4295.
9. Pleguezuelo M, Lopez-Sanchez LM, Rodriguez-Ariza A, Montero JL, Briceno J, Ciria R, Muntane J, de la Mata M: **Proteomic analysis for developing new biomarkers of hepatocellular carcinoma**. *World J Hepatol* 2010, **2**(3):127-135.
10. Sugimoto K, Shiraki K, Takei Y, Ito M, Nobori T, Suzuki H, Dissanayaka SK, Meno K, Asashima M, Uchida K: **Serum protein isoform profiles indicate the progression of hepatitis C virus-induced liver diseases**. *Int J Mol Med* 2013, **31**(4):943-950.
11. Bharali D, Banerjee BD, Bharadwaj M, Husain SA, Kar P: **Expression analysis of apolipoproteins AI & AIV in hepatocellular carcinoma: A protein-based hepatocellular carcinoma-associated study**. *Indian J Med Res* 2018, **147**(4):361-368.
12. Son KH, Ahn CB, Kim HJ, Kim JS: **Quantitative proteomic analysis of bile in extrahepatic cholangiocarcinoma patients**. *J Cancer* 2020, **11**(14):4073-4080.
13. Abulaizi M, Tomonaga T, Satoh M, Sogawa K, Matsushita K, Kodera Y, Obul J, Takano S, Yoshitomi H, Miyazaki M *et al*: **The application of a three-step proteome analysis for identification of new biomarkers of pancreatic cancer**. *Int J Proteomics* 2011, **2011**:628787.
14. Park J, Lee E, Park KJ, Park HD, Kim JW, Woo HI, Lee KH, Lee KT, Lee JK, Park JO *et al*: **Large-scale clinical validation of biomarkers for pancreatic cancer using a mass spectrometry-based proteomics approach**. *Oncotarget* 2017, **8**:42761-42771.
15. Peng H, Pan S, Yan Y, Brand RE, Petersen GM, Chari ST, Lai LA, Eng JK, Brentnall TA, Chen R: **Systemic Proteome Alterations Linked to Early Stage Pancreatic Cancer in Diabetic Patients**. *Cancers (Basel)* 2020, **12**(6):1534.

16. Liu W, Liu B, Cai Q, Li J, Chen X, Zhu Z: **Proteomic identification of serum biomarkers for gastric cancer using multi-dimensional liquid chromatography and 2D differential gel electrophoresis**. *Clin Chim Acta* 2012, **413**(13-14):1098-1106.

17. Sugimachi K, Yamaguchi R, Eguchi H, Ueda M, Niida A, Sakimura S, Hirata H, Uchi R, Shinden Y, Iguchi T *et al*: **8q24 Polymorphisms and Diabetes Mellitus Regulate Apolipoprotein A-IV in Colorectal Carcinogenesis**. *Ann Surg Oncol* 2016, **23**(Suppl 4):546-551.

18. Ahn SB, Sharma S, Mohamedali A, Mahboob S, Redmond WJ, Pascovici D, Wu JX, Zaw T, Adhikari S, Vaibhav V *et al*: **Potential early clinical stage colorectal cancer diagnosis using a proteomics blood test panel**. *Clin Proteomics* 2019, **16**:34.

19. Karczmarski J, Rubel T, Mikula M, Wolski J, Rutkowski A, Zagorowicz E, Dadlez M, Ostrowski J: **Pre-analytical-related variability influencing serum peptide profiles demonstrated in a mass spectrometry-based search for colorectal and prostate cancer biomarkers**. *Acta Biochimica Polnica* 2013, **60**:417-425.

20. Chang SC, Lin WL, Chang YF, Lee CT, Wu JS, Hsu PH, Chang CF: **Glycoproteomic identification of novel plasma biomarkers for oral cancer**. *J Food Drug Anal* 2019, **27**(2):483-493.

21. Abdullah MI, Lee CC, Mat Junit S, Ng KL, Hashim OH: **Tissue and serum samples of patients with papillary thyroid cancer with and without benign background demonstrate different altered expression of proteins**. *PeerJ* 2016, **4**:e2450.

22. Farrokhi Yekta R, Arefi Oskouie A, Rezaei Tavirani M, Mohajeri-Tehrani MR, Soroush AR: **Decreased apolipoprotein A4 and increased complement component 3 as potential markers for papillary thyroid carcinoma: A proteomic study**. *Int J Biol Markers* 2018, **33**(4):455-462.

23. Li D, Wu J, Liu Z, Qiu L, Zhang Y: **Novel circulating protein biomarkers for thyroid cancer determined through data-independent acquisition mass spectrometry**. *PeerJ* 2020, **8**:e9507.

24. Dowling P, O'Driscoll L, Meleady P, Henry M, Roy S, Ballot J, Moriarty M, Crown J, Clynes M: **2-D difference gel electrophoresis of the lung squamous cell carcinoma versus normal sera demonstrates consistent alterations in the levels of ten specific proteins**. *Electrophoresis* 2007, **28**(23):4302-4310.

25. Okano T, Seike M, Kuribayashi H, Soeno C, Ishii T, Kida K, Gemma A: **Identification of haptoglobin peptide as a novel serum biomarker for lung squamous cell carcinoma by serum proteome and peptidome profiling**. *Int J Oncol* 2016, **48**(3):945-952.

26. Zheng RJ, Wu RJ, Ma XD: **Serum proteomic spectral characteristics of acute myeloid leukemia and their clinical significance**. *Genet Mol Res* 2017, **16**.

27. Miyauchi E, Furuta T, Ohtsuki S, Tachikawa M, Uchida Y, Sabit H, Obuchi W, Baba T, Watanabe M, Terasaki T *et al*: **Identification of blood biomarkers in glioblastoma by SWATH mass spectrometry and quantitative targeted absolute proteomics**. *PLoS ONE* 2018, **13**:e0193799.

28. Soukup V, Capoun O, Pesl M, Vavrova L, Sobotka R, Levova K, Hanus T, Zima T, Kalousova M: **The significance of calprotectin, CD147, APOA4 and DJ-1 in non-invasive detection of urinary bladder carcinoma**. *Neoplasma* 2019, **66**(6):1019-1023.

29. Levey AS, Inker LA, Coresh J: **GFR estimation: from physiology to public health**. *Am J Kidney Dis* 2014, **63**(5):820-834.
